# Supplementary material for: p300/CBP degradation is required to disable the active AR enhanceosome in prostate cancer
Source: bioRxiv. 2024 May 23:2024.03.29.587346. Originally published 2024 Mar 30. Preprint. [Version 2] doi: 10.1101/2024.03.29.587346 (PMC10996709; doi:10.1101/2024.03.29.587346)
Supplement: Supplement 1 — Figure S1: p300 is the critical histone acetyltransferase for H2BNTac and assessment of the active ERG cistrome in prostate cancer. a. Immunoblot analysis of key histone marks in four pairs of matched prostate cancer (T) and benign adjacent tissues (N). Quantitation and fold change (FC) of the respective histone marks is provided to the right. PTMs, post-translational modifications. b. Representative immunofluorescence images of H2BK5ac (green) and H2BK20ac (green) expression in nuclei of paired prostate cancer and benign adjacent tissues. Magnification: 200x. Scalebar = 50 μm. c. Venn diagrams illustrating overlaps of genome-wide p300, H2BK5ac, H2BK20ac, H3K18ac, and H3K27ac ChIP-seq peaks in VCaP cells. d. Bar charts depicting proportion of FOXA1, SMARCA4, p300, and BRD4 ChIP-seq peaks that map to all AR peaks located in non-promoter regions. e. Bar charts depicting proportion of FOXA1, SMARCA4, p300, and BRD4 peaks that map to the top quartile of ERG ChIP-seq peaks located in non-promoter regions. f. ChIP-seq (ERG, FOXA1, p300, MED1, H3K27ac, and H2BK20ac) and ATAC-seq read-density heatmaps at ERG/p300 co-bound and ERG only binding sites in VCaP cells. g. Immunoblot analysis of p300, CBP, and indicated histone marks in 22Rv1 WT, p300 KO, CBP KO, and p300 KO with siCBP cells. Figure S2: Characterization of the on-target degradation effects of the p300/CBP degrader, CBPD-409. a. ChIP-seq read-density heatmaps of H3K27ac at AR/p300 co-bound sites in VCaP cells with 4 hours of 1 μM GNE-049 or 1 μM CCS1477 treatments. b. Immunoblot analysis of p300 and CBP in LNCaP cells treated with 100 nM CBPD-409 for the indicated durations. c. Immunoblot analysis of p300 and CBP in VCaP cells treated with 10 nM or 100 nM CBPD-409 for 4 hours. d. Immunoblot analysis of p300 and CBP in non-neoplastic prostatic cells treated with 10 nM or 100 nM CBPD-409 for 4 hours. e. Immunoblot analysis of p300 and CBP in murine prostate cancer cells (Myc-Cap and TRAMPC2) treated with 10 nM or 100 [file media-1.pdf]

Figure S1

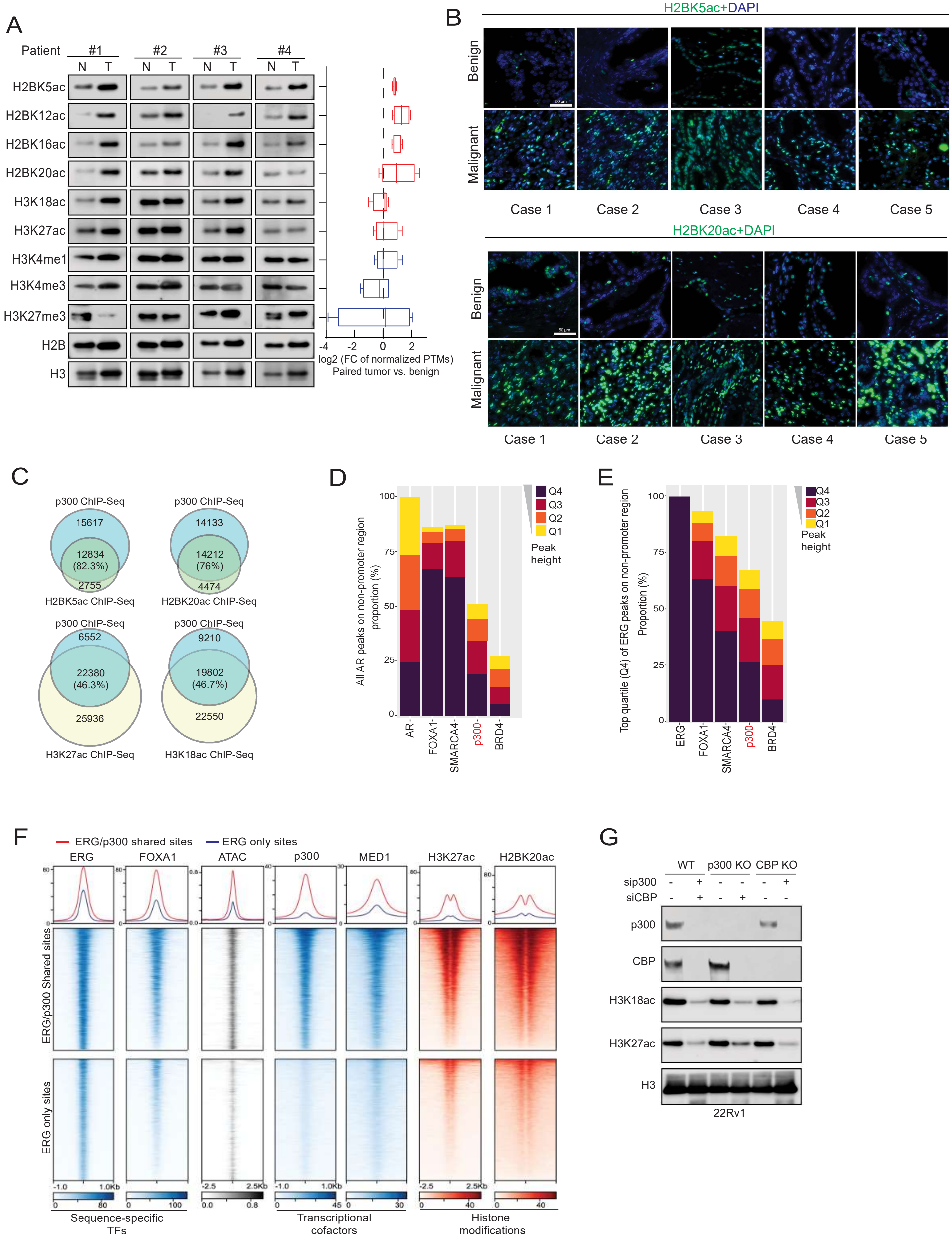

A
Patient #1 #2 #3 #4
N T N T N T N T
H2BK5ac
H2BK12ac
H2BK16ac
H2BK20ac
H3K18ac
H3K27ac
H3K4me1
H3K4me3
H3K27me3
H2B
H3
-4 -2 0 2
log2 (FC of normalized PTMs)
Paired tumor vs. benign
B
H2BK5ac+DAPI
Benign
Malignant
Case 1 Case 2 Case 3 Case 4 Case 5
H2BK20ac+DAPI
Benign
Malignant
Case 1 Case 2 Case 3 Case 4 Case 5
C
p300 ChIP-Seq
15617
12834 (82.3%)
2755
H2BK5ac ChIP-Seq
p300 ChIP-Seq
14133
14212 (76%)
4474
H2BK20ac ChIP-Seq
p300 ChIP-Seq
6552
22380 (46.3%)
25936
H3K27ac ChIP-Seq
p300 ChIP-Seq
9210
19802 (46.7%)
22550
H3K18ac ChIP-Seq
D
All AR peaks on non-promoter region proportion (%)
0 25 50 75 100
AR FOXA1 SMARCA4 p300 BRD4
Q4 Q3 Q2 Q1
Peak height
E
Top quartile (Q4) of ERG peaks on non-promoter region Proportion (%)
0 25 50 75 100
ERG FOXA1 SMARCA4 p300 BRD4
Q4 Q3 Q2 Q1
Peak height
F
ERG/p300 shared sites
ERG only sites
ERG FOXA1 ATAC p300 MED1 H3K27ac H2BK20ac
ERG/p300 Shared sites
ERG only sites
-1.0 1.0Kb
-2.5 2.5Kb
-1.0 1.0Kb
-2.5 2.5Kb
0 80
0 100
0.0 0.8
0 45
0 30
0 40
0 40
Sequence-specific TFs
Transcriptional cofactors
Histone modifications
G
WT p300 KO CBP KO
sip300 - + - - - +
siCBP - + - + - -
p300
CBP
H3K18ac
H3K27ac
H3
22Rv1

Figure S2

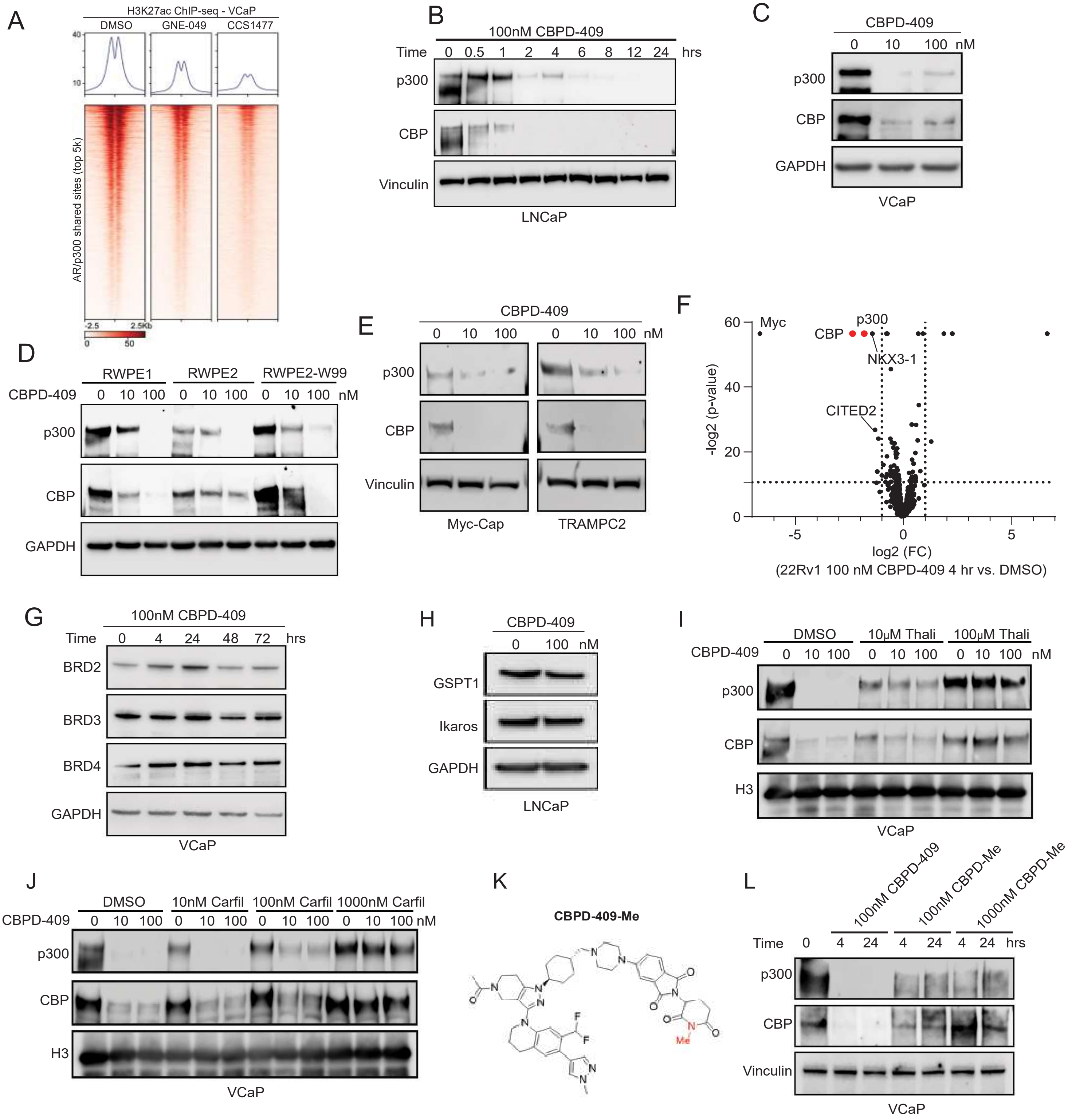
A
H3K27ac ChIP-seq - VCaP
DMSO
GNE-049
CCS1477
AR/p300 shared sites (top 5k)
-2.5
2.5Kb
B
100nM CBPD-409
Time 0 0.5 1 2 4 6 8 12 24 hrs
p300
CBP
Vinculin
LNCaP
C
CBPD-409
0 10 100 nM
p300
CBP
GAPDH
VCaP
D
RWPE1
RWPE2
RWPE2-W99
CBPD-409 0 10 100 0 10 100 0 10 100 nM
p300
CBP
GAPDH
E
CBPD-409
0 10 100 0 10 100 nM
p300
CBP
Vinculin
Myc-Cap
TRAMPC2
F
Myc
p300
CBP
NKX3-1
CITED2
-log2 (p-value)
log2 (FC)
(22Rv1 100 nM CBPD-409 4 hr vs. DMSO)
G
100nM CBPD-409
Time 0 4 24 48 72 hrs
BRD2
BRD3
BRD4
GAPDH
VCaP
H
CBPD-409
0 100 nM
GSPT1
Ikaros
GAPDH
LNCaP
I
DMSO
10μM Thali
100μM Thali
CBPD-409 0 10 100 0 10 100 0 10 100 nM
p300
CBP
H3
VCaP
J
DMSO
10nM Carfil
100nM Carfil
1000nM Carfil
CBPD-409 0 10 100 0 10 100 0 10 100 0 10 100 nM
p300
CBP
H3
VCaP
K
CBPD-409-Me
L
100nM CBPD-409
100nM CBPD-Me
1000nM CBPD-Me
Time 0 4 24 4 24 4 24 hrs
p300
CBP
Vinculin
VCaP

Figure S3

A

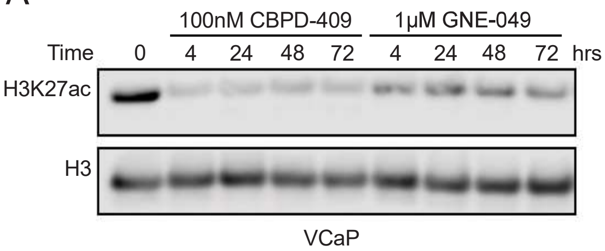

B

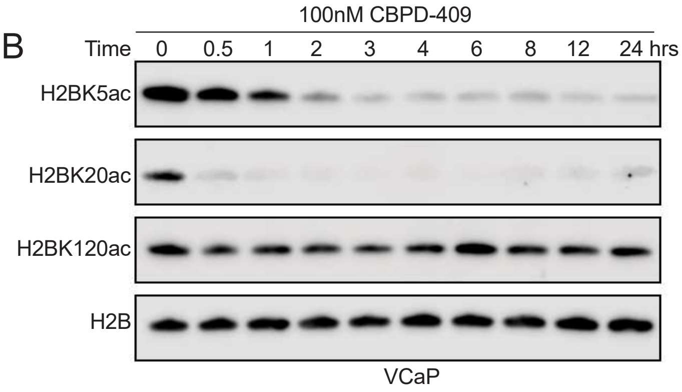

C

Summary of differential acetylated sites (acetylated proteins)

Group Name	Up-regulated (FC>1.5, p<0.05)	Down-regulated (FC<1/1.5, p<0.05)
DMSO vs CBPD 1hr	16 (11)	134 (80)
DMSO vs CBPD 4hrs	4 (2)	243 (151)
DMSO vs CBPD 12hrs	5 (5)	302 (197)
DMSO vs CBPD 24hrs	11 (8)	337 (214)

D

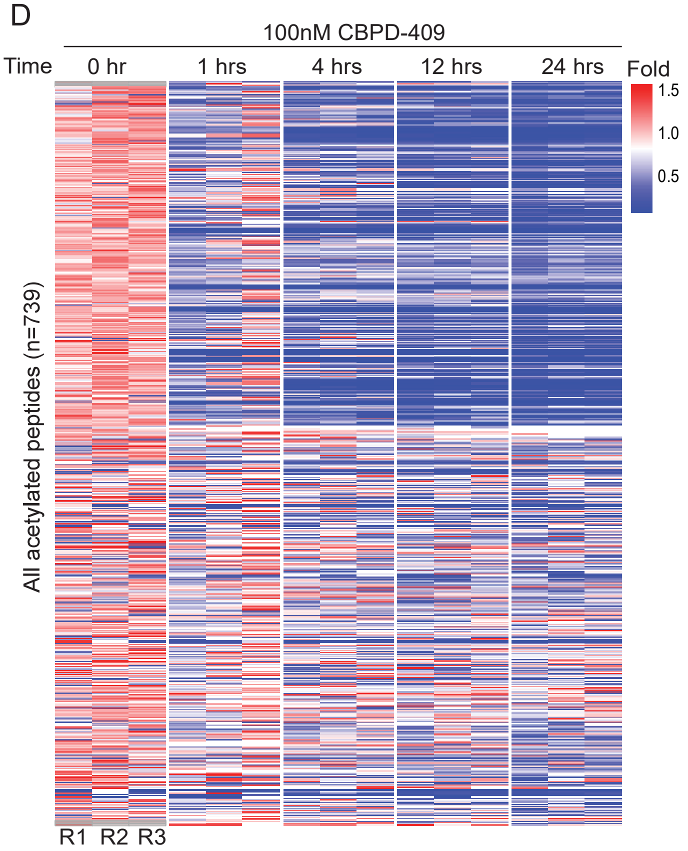

E

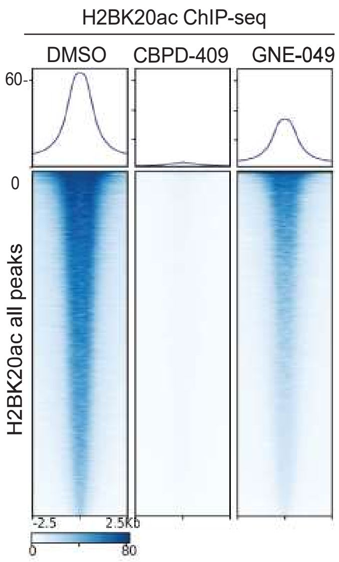

Figure S4

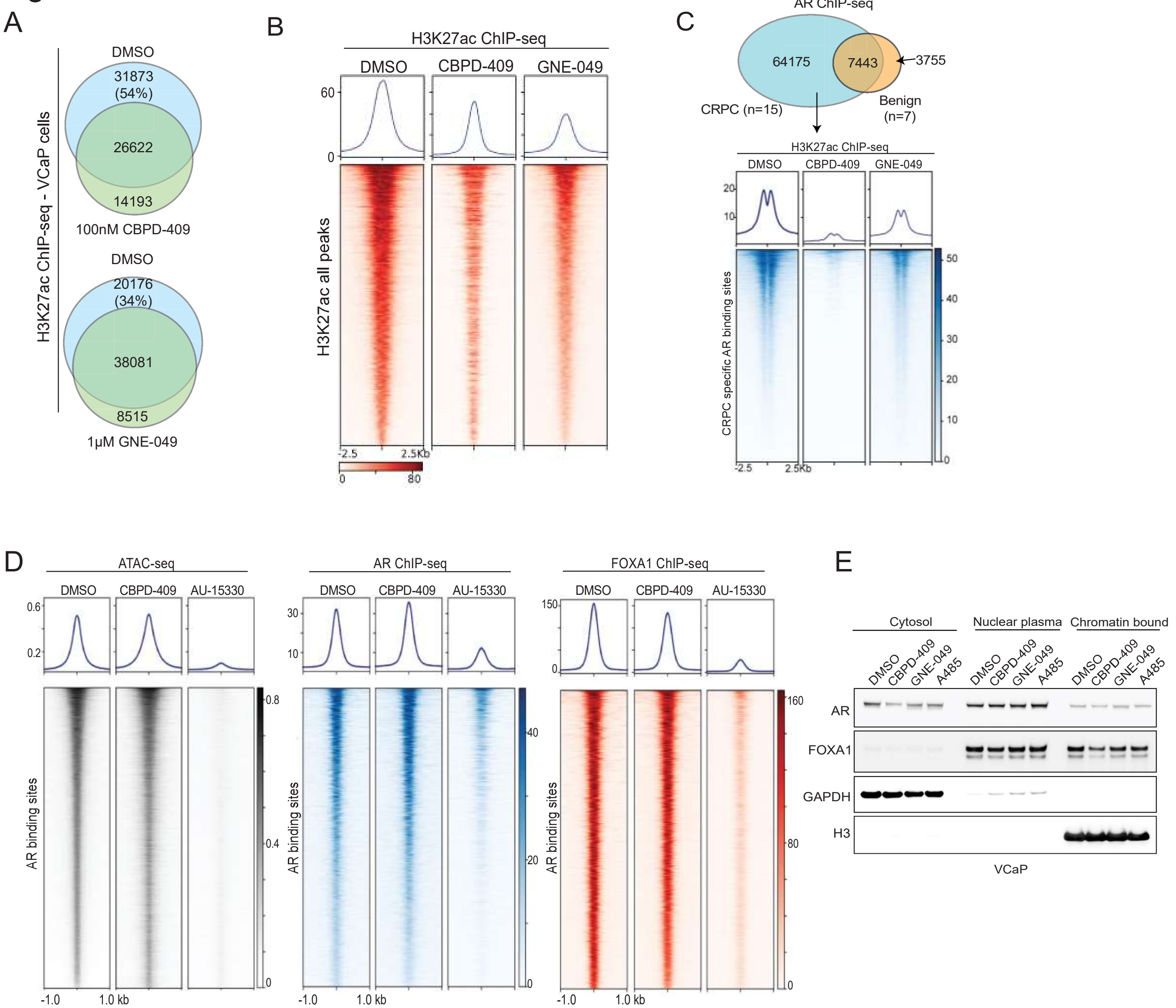

A
H3K27ac ChIP-seq - VCaP cells
DMSO
31873 (54%)
26622
14193
100nM CBPD-409
DMSO
20176 (34%)
38081
8515
1μM GNE-049
B
H3K27ac ChIP-seq
DMSO
CBPD-409
GNE-049
H3K27ac all peaks
C
AR ChIP-seq
64175
7443
3755
CRPC (n=15)
Benign (n=7)
H3K27ac ChIP-seq
DMSO
CBPD-409
GNE-049
CRPC specific AR binding sites
D
ATAC-seq
AR ChIP-seq
FOXA1 ChIP-seq
DMSO
CBPD-409
AU-15330
AR binding sites
E
Cytosol
Nuclear plasma
Chromatin bound
DMSO
CBPD-409
GNE-049
A485
AR
FOXA1
GAPDH
H3
VCaP

Figure S5

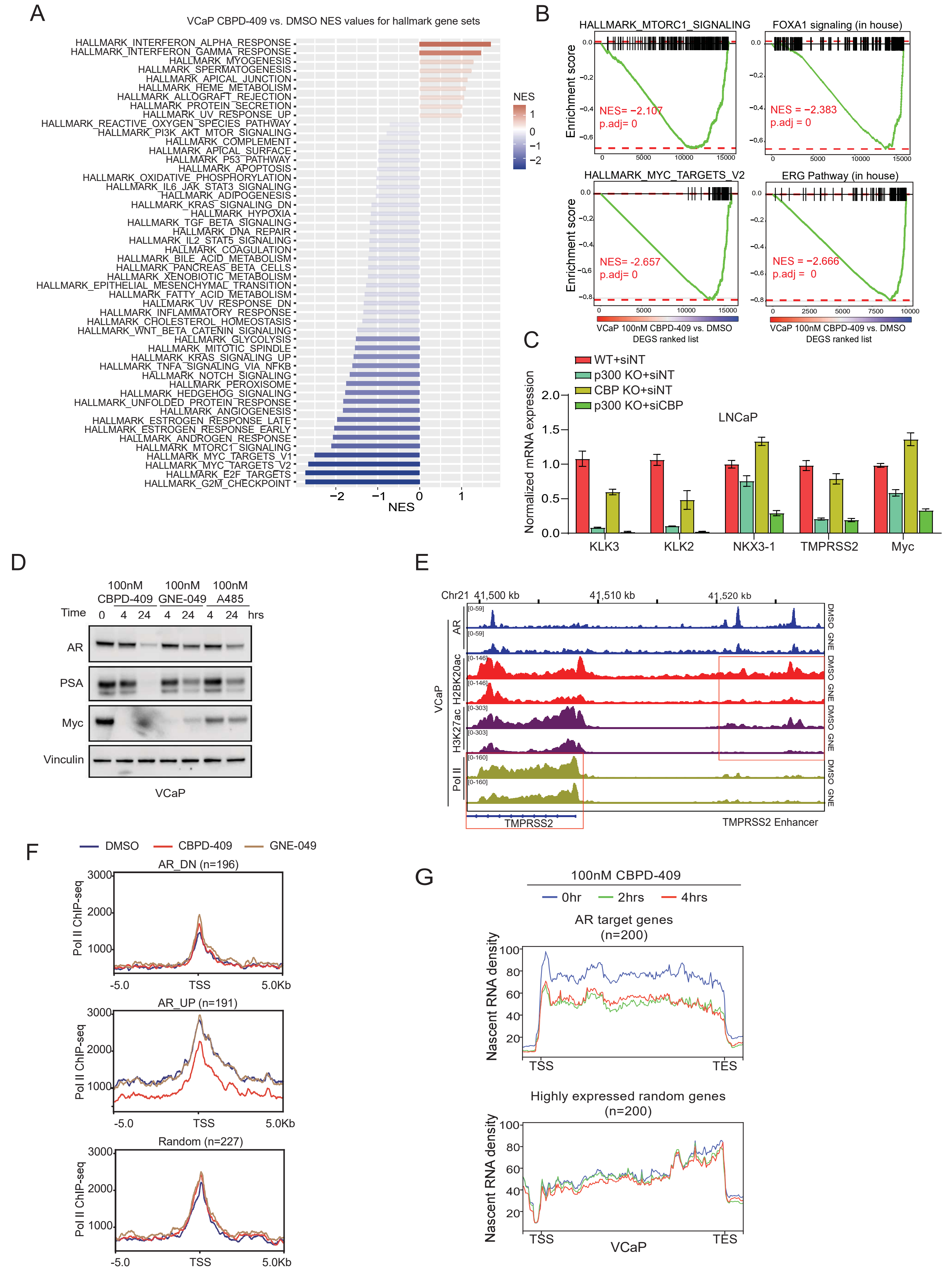

A
VCaP CBPD-409 vs. DMSO NES values for hallmark gene sets
HALLMARK_INTERFERON_ALPHA_RESPONSE
HALLMARK_INTERFERON_GAMMA_RESPONSE
HALLMARK_MYOGENESIS
HALLMARK_SPERMATOGENESIS
HALLMARK_APICAL_JUNCTION
HALLMARK_HEME_METABOLISM
HALLMARK_ALLOGRAFT_REJECTION
HALLMARK_PROTEIN_SECRETION
HALLMARK_UV_RESPONSE_UP
HALLMARK_REACTIVE_OXYGEN_SPECIES_PATHWAY
HALLMARK_PI3K_AKT_MTOR_SIGNALING
HALLMARK_COMPLEMENT
HALLMARK_APICAL_SURFACE
HALLMARK_P53_PATHWAY
HALLMARK_APOPTOSIS
HALLMARK_OXIDATIVE_PHOSPHORYLATION
HALLMARK_IL6_JAK_STAT3_SIGNALING
HALLMARK_ADIPOGENESIS
HALLMARK_KRAS_SIGNALING_DN
HALLMARK_HYPOXIA
HALLMARK_TGF_BETA_SIGNALING
HALLMARK_DNA_REPAIR
HALLMARK_IL2_STAT5_SIGNALING
HALLMARK_COAGULATION
HALLMARK_BILE_ACID_METABOLISM
HALLMARK_PANCREAS_BETA_CELLS
HALLMARK_XENOBIOTIC_METABOLISM
HALLMARK_EPITHELIAL_MESENCHYMAL_TRANSITION
HALLMARK_FATTY_ACID_METABOLISM
HALLMARK_UV_RESPONSE_DN
HALLMARK_INFLAMMATORY_RESPONSE
HALLMARK_CHOLESTEROL_HOMEOSTASIS
HALLMARK_WNT_BETA_CATENIN_SIGNALING
HALLMARK_GLYCOLYSIS
HALLMARK_MITOTIC_SPINDLE
HALLMARK_KRAS_SIGNALING_UP
HALLMARK_TNFA_SIGNALING_VIA_NFKB
HALLMARK_NOTCH_SIGNALING
HALLMARK_PEROXISOME
HALLMARK_HEDGEHOG_SIGNALING
HALLMARK_UNFOLDED_PROTEIN_RESPONSE
HALLMARK_ANGIOGENESIS
HALLMARK_ESTROGEN_RESPONSE_LATE
HALLMARK_ESTROGEN_RESPONSE_EARLY
HALLMARK_ANDROGEN_RESPONSE
HALLMARK_MTORC1_SIGNALING
HALLMARK_MYC_TARGETS_V1
HALLMARK_MYC_TARGETS_V2
HALLMARK_E2F_TARGETS
HALLMARK_G2M_CHECKPOINT
−2 −1 0 1
NES
NES
1
0
−1
−2
B
HALLMARK_MTORC1_SIGNALING
FOXA1 signaling (in house)
Enrichment score
NES= −2.107
p.adj= 0
NES = −2.383
p.adj = 0
HALLMARK_MYC_TARGETS_V2
ERG Pathway (in house)
NES= -2.657
p.adj= 0
NES = −2.666
p.adj = 0
VCaP 100nM CBPD-409 vs. DMSO
DEGS ranked list
VCaP 100nM CBPD-409 vs. DMSO
DEGS ranked list
C
WT+siNT
p300 KO+siNT
CBP KO+siNT
p300 KO+siCBP
LNCaP
Normalized mRNA expression
KLK3
KLK2
NKX3-1
TMPRSS2
Myc
D
100nM CBPD-409
100nM GNE-049
100nM A485
Time 0 4 24 4 24 4 24 hrs
AR
PSA
Myc
Vinculin
VCaP
E
Chr21 41,500 kb
41,510 kb
41,520 kb
VCaP
AR
H2BK20ac
H3K27ac
Pol II
DMSO
GNE
[0-59]
[0-146]
[0-303]
[0-160]
TMPRSS2
TMPRSS2 Enhancer
F
DMSO
CBPD-409
GNE-049
AR_DN (n=196)
AR_UP (n=191)
Random (n=227)
Pol II ChIP-seq
-5.0
TSS
5.0Kb
G
100nM CBPD-409
0hr
2hrs
4hrs
AR target genes
(n=200)
Highly expressed random genes
(n=200)
Nascent RNA density
TSS
TES
VCaP

Figure S6

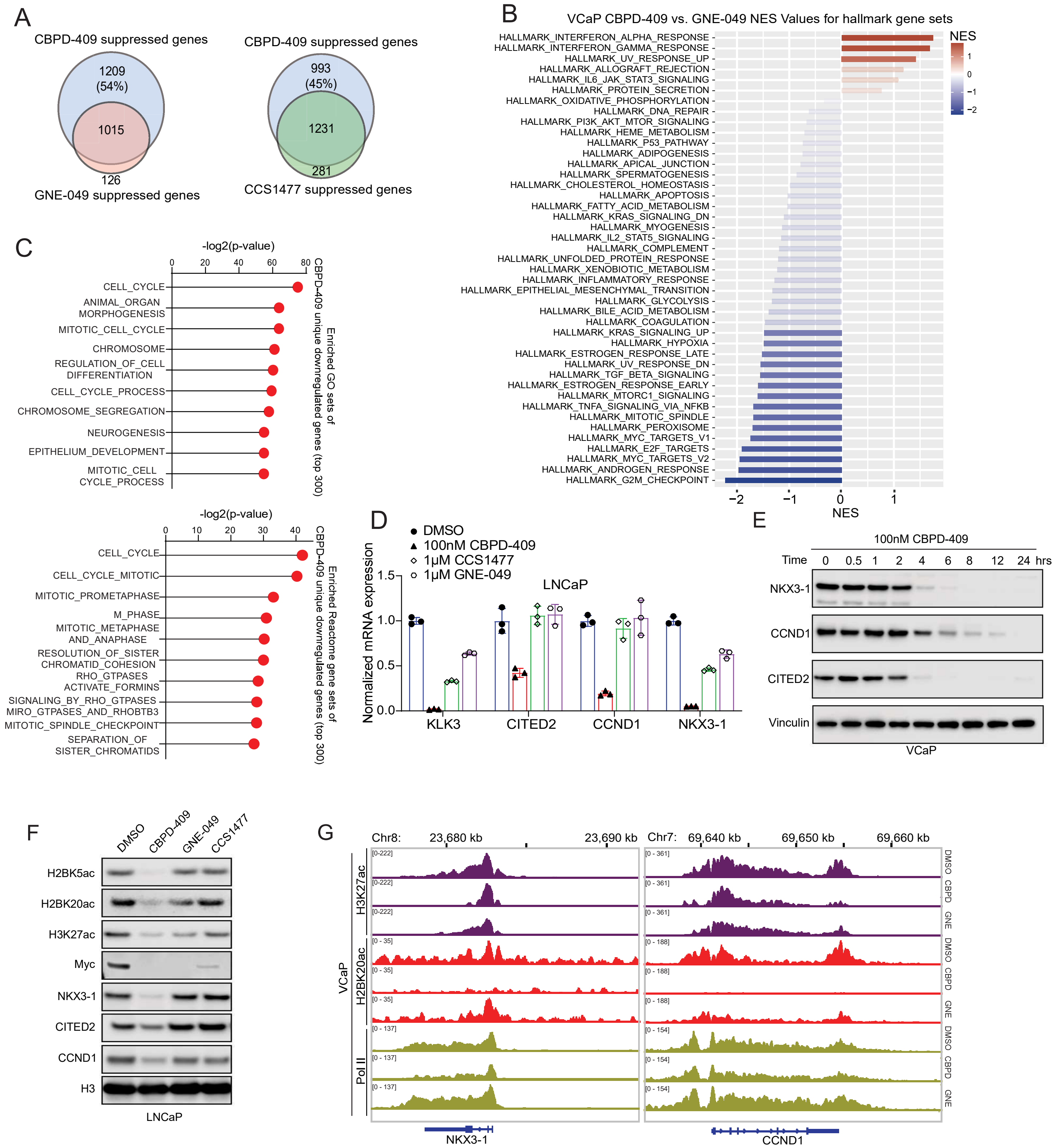

A
CBPD-409 suppressed genes
1209
(54%)
1015
126
GNE-049 suppressed genes
CBPD-409 suppressed genes
993
(45%)
1231
281
CCS1477 suppressed genes
B
VCaP CBPD-409 vs. GNE-049 NES Values for hallmark gene sets
HALLMARK_INTERFERON_ALPHA_RESPONSE
HALLMARK_INTERFERON_GAMMA_RESPONSE
HALLMARK_UV_RESPONSE_UP
HALLMARK_ALLOGRAFT_REJECTION
HALLMARK_IL6_JAK_STAT3_SIGNALING
HALLMARK_PROTEIN_SECRETION
HALLMARK_OXIDATIVE_PHOSPHORYLATION
HALLMARK_DNA_REPAIR
HALLMARK_PI3K_AKT_MTOR_SIGNALING
HALLMARK_HEME_METABOLISM
HALLMARK_P53_PATHWAY
HALLMARK_ADIPOGENESIS
HALLMARK_APICAL_JUNCTION
HALLMARK_SPERMATOGENESIS
HALLMARK_CHOLESTEROL_HOMEOSTASIS
HALLMARK_APOPTOSIS
HALLMARK_FATTY_ACID_METABOLISM
HALLMARK_KRAS_SIGNALING_DN
HALLMARK_MYOGENESIS
HALLMARK_IL2_STAT5_SIGNALING
HALLMARK_COMPLEMENT
HALLMARK_UNFOLDED_PROTEIN_RESPONSE
HALLMARK_XENOBIOTIC_METABOLISM
HALLMARK_INFLAMMATORY_RESPONSE
HALLMARK_EPITHELIAL_MESENCHYMAL_TRANSITION
HALLMARK_GLYCOLYSIS
HALLMARK_BILE_ACID_METABOLISM
HALLMARK_COAGULATION
HALLMARK_KRAS_SIGNALING_UP
HALLMARK_HYPOXIA
HALLMARK_ESTROGEN_RESPONSE_LATE
HALLMARK_UV_RESPONSE_DN
HALLMARK_TGF_BETA_SIGNALING
HALLMARK_ESTROGEN_RESPONSE_EARLY
HALLMARK_MTORC1_SIGNALING
HALLMARK_TNFA_SIGNALING_VIA_NFKB
HALLMARK_MITOTIC_SPINDLE
HALLMARK_PEROXISOME
HALLMARK_MYC_TARGETS_V1
HALLMARK_E2F_TARGETS
HALLMARK_MYC_TARGETS_V2
HALLMARK_ANDROGEN_RESPONSE
HALLMARK_G2M_CHECKPOINT
−2 −1 0 1
NES
NES
1
0
−1
−2
C
-log2(p-value)
0 20 40 60 80
CELL_CYCLE
ANIMAL_ORGAN MORPHOGENESIS
MITOTIC_CELL_CYCLE
CHROMOSOME
REGULATION_OF_CELL DIFFERENTIATION
CELL_CYCLE_PROCESS
CHROMOSOME_SEGREGATION
NEUROGENESIS
EPITHELIUM_DEVELOPMENT
MITOTIC_CELL CYCLE_PROCESS
Enriched GO sets of CBPD-409 unique downregulated genes (top 300)
-log2(p-value)
0 10 20 30 40
CELL_CYCLE
CELL_CYCLE_MITOTIC
MITOTIC_PROMETAPHASE
M_PHASE
MITOTIC_METAPHASE AND_ANAPHASE
RESOLUTION_OF_SISTER CHROMATID_COHESION
RHO_GTPASES ACTIVATE_FORMINS
SIGNALING_BY_RHO_GTPASES MIRO_GTPASES_AND_RHOBTB3
MITOTIC_SPINDLE_CHECKPOINT
SEPARATION_OF SISTER_CHROMATIDS
Enriched Reactome gene sets of CBPD-409 unique downregulated genes (top 300)
D
DMSO
100nM CBPD-409
1μM CCS1477
1μM GNE-049
LNCaP
Normalized mRNA expression
0.0 0.5 1.0 1.5
KLK3
CITED2
CCND1
NKX3-1
E
100nM CBPD-409
Time 0 0.5 1 2 4 6 8 12 24 hrs
NKX3-1
CCND1
CITED2
Vinculin
VCaP
F
DMSO
CBPD-409
GNE-049
CCS1477
H2BK5ac
H2BK20ac
H3K27ac
Myc
NKX3-1
CITED2
CCND1
H3
LNCaP
G
Chr8: 23,680 kb 23,690 kb
Chr7: 69,640 kb 69,650 kb 69,660 kb
VCaP
H3K27ac
H2BK20ac
Pol II
[0-222]
[0 - 35]
[0 - 137]
[0 - 361]
[0 - 188]
[0 - 154]
DMSO
CBPD
GNE
NKX3-1
CCND1

Figure S7

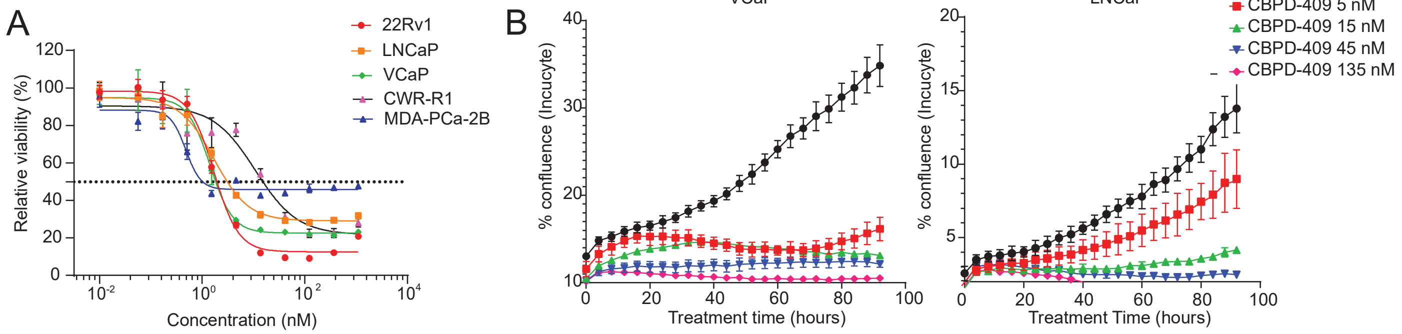

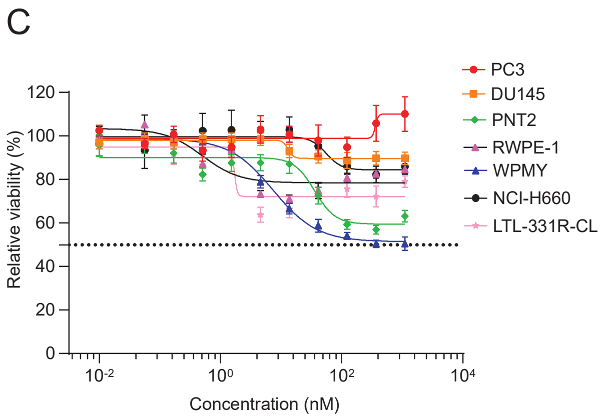

D

	Cell line	CBPD-409 IC50 (nM)
AR+ prostate cancer	22Rv1	2.2
	MDA-PCA-2B	2.5
	VCaP	4
	LNCaP	11
	CWR-R1	11.5
AR- prostate cancer	PC3	6368
	DU145	>30000
	NCI-H660	>30000
	LTL-331R-CL	>30000
non-neoplastic	WPMY-1	4365
	PNT2	>30000
	RWPE-1	>30000

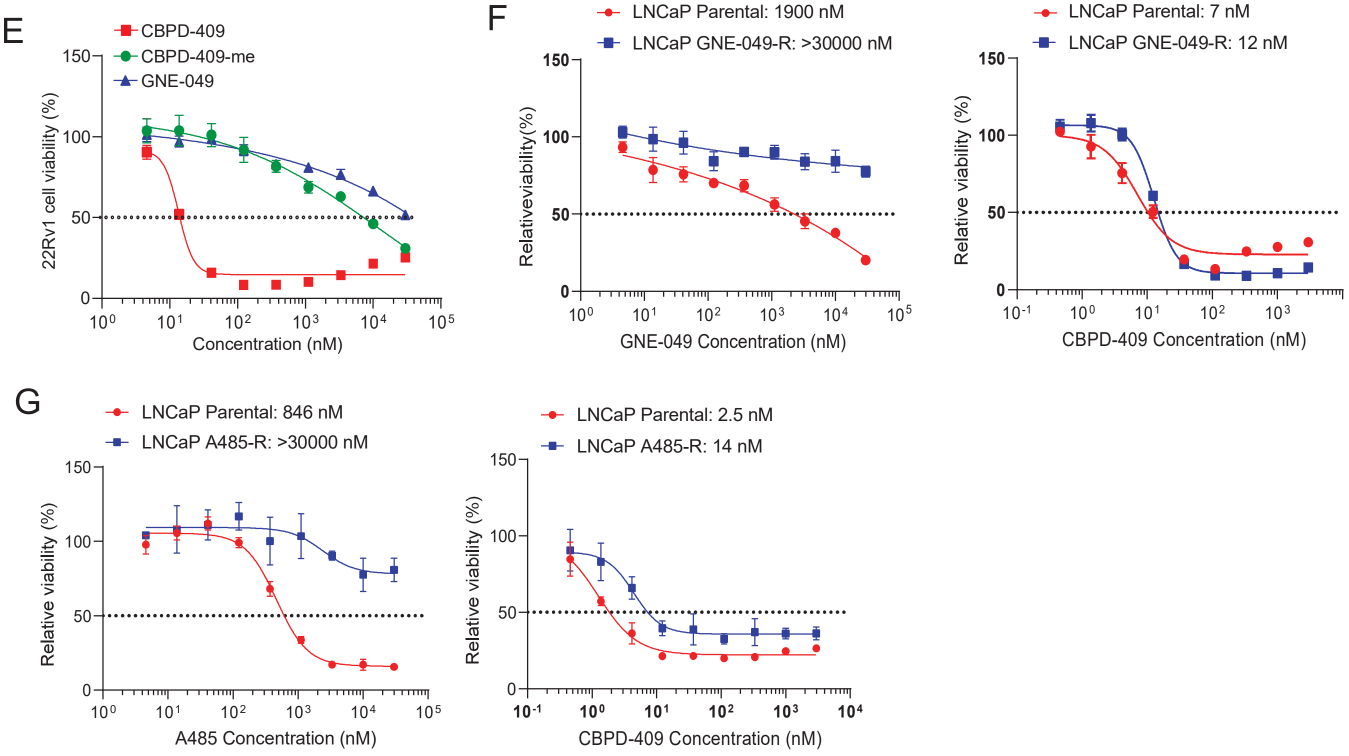

Figure S8

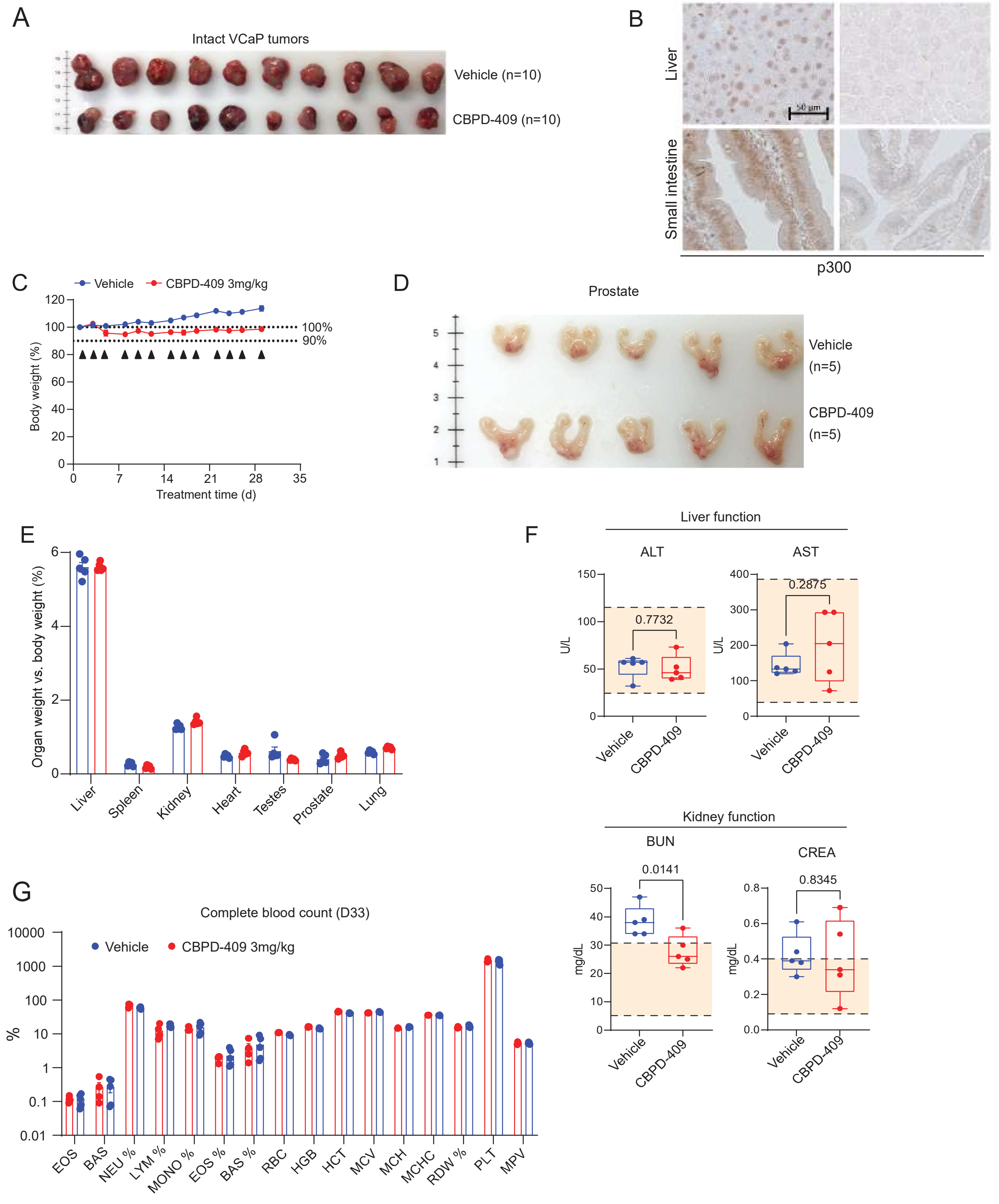
A
Intact VCaP tumors
Vehicle (n=10)
CBPD-409 (n=10)
B
Vehicle
CBPD-409
Liver
Small intestine
p300
C
Vehicle
CBPD-409 3mg/kg
Body weight (%)
100%
90%
Treatment time (d)
D
Prostate
Vehicle (n=5)
CBPD-409 (n=5)
E
Organ weight vs. body weight (%)
Liver
Spleen
Kidney
Heart
Testes
Prostate
Lung
F
Liver function
ALT
AST
U/L
0.7732
0.2875
Vehicle
CBPD-409
Kidney function
BUN
CREA
mg/dL
0.0141
0.8345
G
Complete blood count (D33)
Vehicle
CBPD-409 3mg/kg
%
EOS
BAS
NEU %
LYM %
MONO %
EOS %
BAS %
RBC
HGB
HCT
MCV
MCH
MCHC
RDW %
PLT
MPV

Figure S9

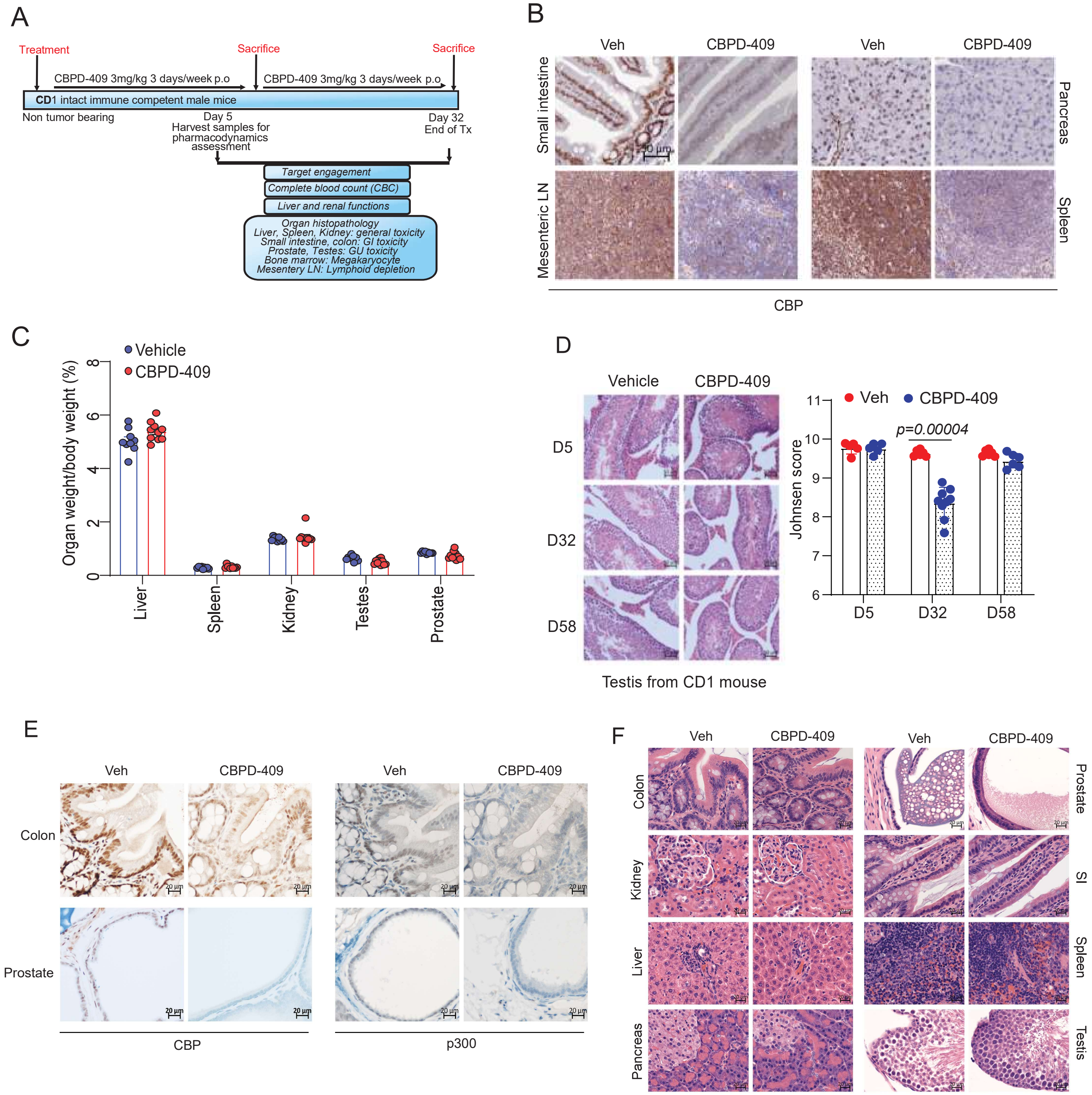
A
Treatment
Sacrifice
Sacrifice
CBPD-409 3mg/kg 3 days/week p.o
CBPD-409 3mg/kg 3 days/week p.o
CD1 intact immune competent male mice
Non tumor bearing
Day 5
Harvest samples for pharmacodynamics assessment
Day 32
End of Tx
Target engagement
Complete blood count (CBC)
Liver and renal functions
Organ histopathology
Liver, Spleen, Kidney: general toxicity
Small intestine, colon: GI toxicity
Prostate, Testes: GU toxicity
Bone marrow: Megakaryocyte
Mesentery LN: Lymphoid depletion
B
Veh
CBPD-409
Veh
CBPD-409
Small intestine
Mesenteric LN
Pancreas
Spleen
CBP
C
Vehicle
CBPD-409
Organ weight/body weight (%)
0
2
4
6
8
Liver
Spleen
Kidney
Testes
Prostate
D
Vehicle
CBPD-409
D5
D32
D58
Testis from CD1 mouse
Veh
CBPD-409
p=0.00004
Johnsen score
6
7
8
9
10
11
D5
D32
D58
E
Veh
CBPD-409
Veh
CBPD-409
Colon
Prostate
CBP
p300
F
Veh
CBPD-409
Veh
CBPD-409
Colon
Kidney
Liver
Pancreas
Prostate
SI
Spleen
Testis

Figure S10

A

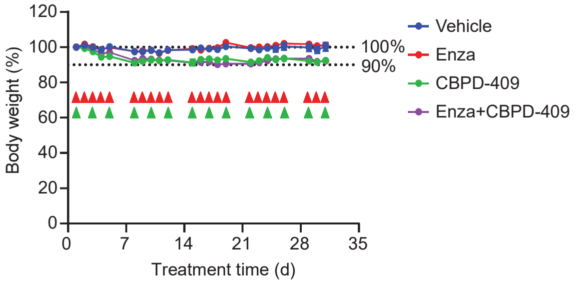

B

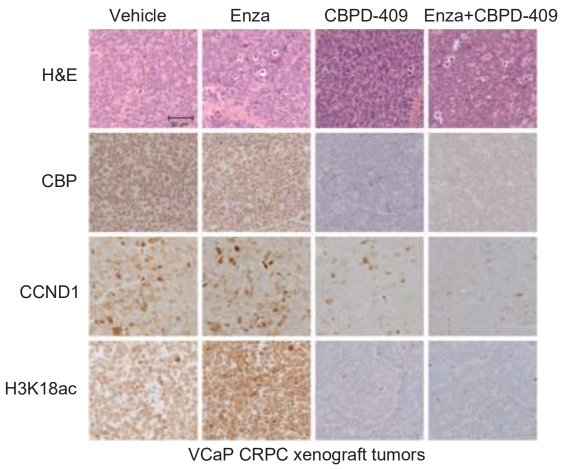

C

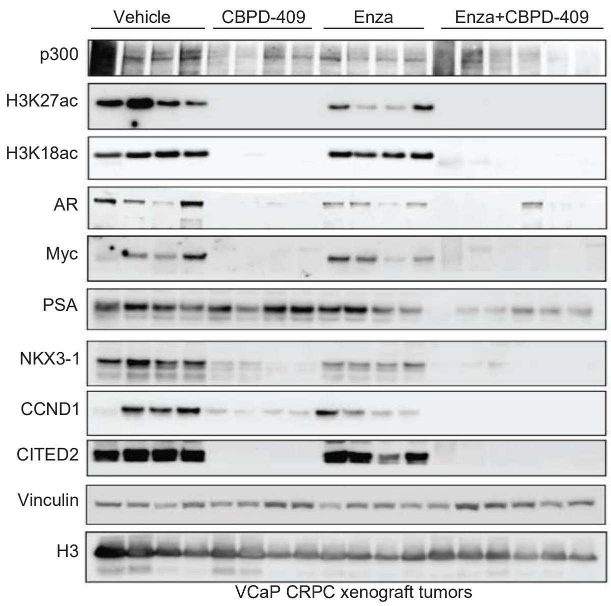

Figure S11

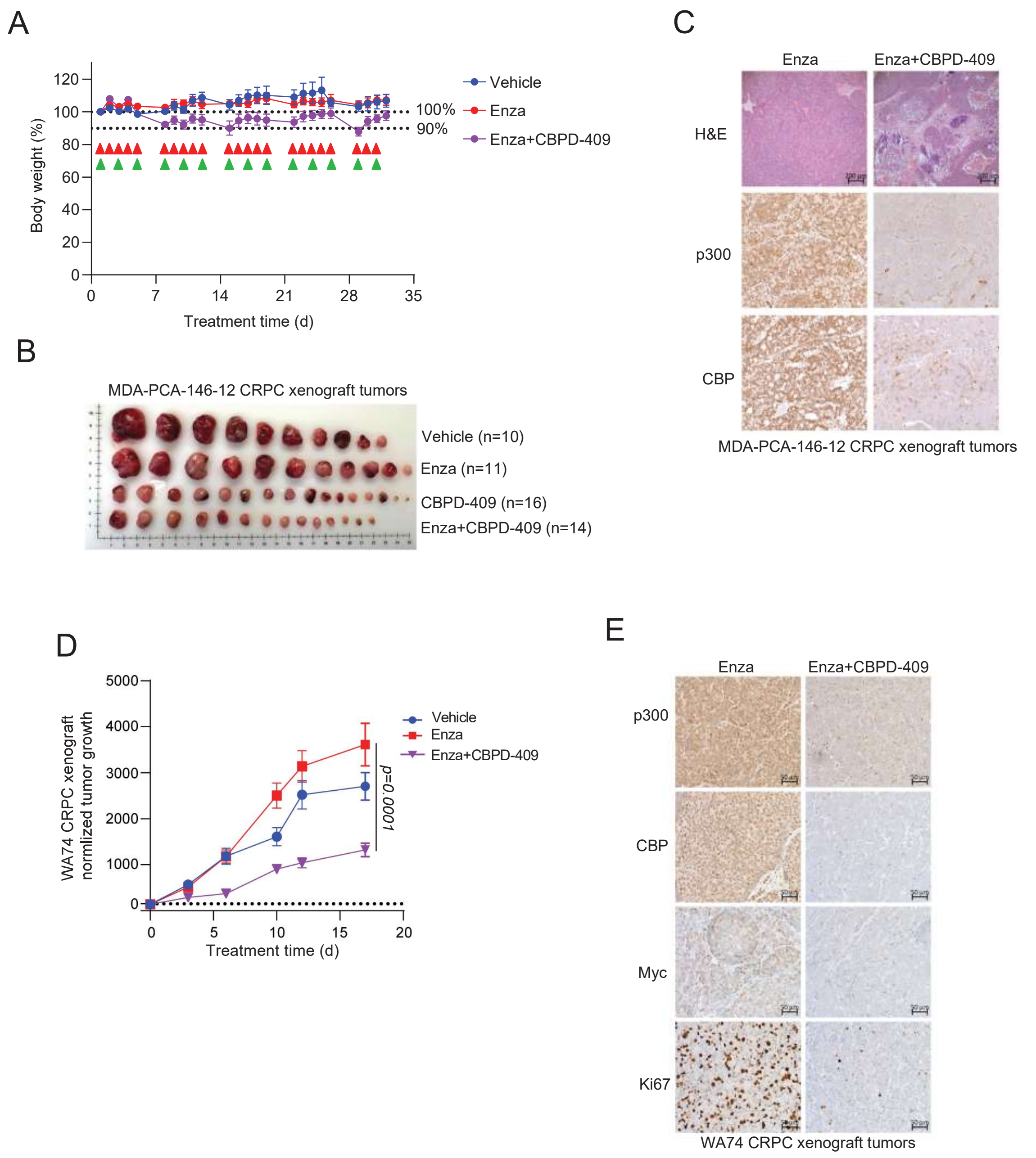

A
120
100
80
60
40
20
0
Body weight (%)
0
7
14
21
28
35
Treatment time (d)
100%
90%
Vehicle
Enza
Enza+CBPD-409
B
MDA-PCA-146-12 CRPC xenograft tumors
Vehicle (n=10)
Enza (n=11)
CBPD-409 (n=16)
Enza+CBPD-409 (n=14)
C
Enza
Enza+CBPD-409
H&E
p300
CBP
MDA-PCA-146-12 CRPC xenograft tumors
D
5000
4000
3000
2000
1000
0
WA74 CRPC xenograft normlized tumor growth
0
5
10
15
20
Treatment time (d)
Vehicle
Enza
Enza+CBPD-409
p=0.0001
E
Enza
Enza+CBPD-409
p300
CBP
Myc
Ki67
WA74 CRPC xenograft tumors
