# Supplementary material for: p300/CBP degradation is required to disable the active AR enhanceosome in prostate cancer
Source: bioRxiv. 2024 Mar 30:2024.03.29.587346. Preprint. [Version 1] doi: 10.1101/2024.03.29.587346 (PMC10996709; doi:10.1101/2024.03.29.587346)

Extended Data Fig. 1

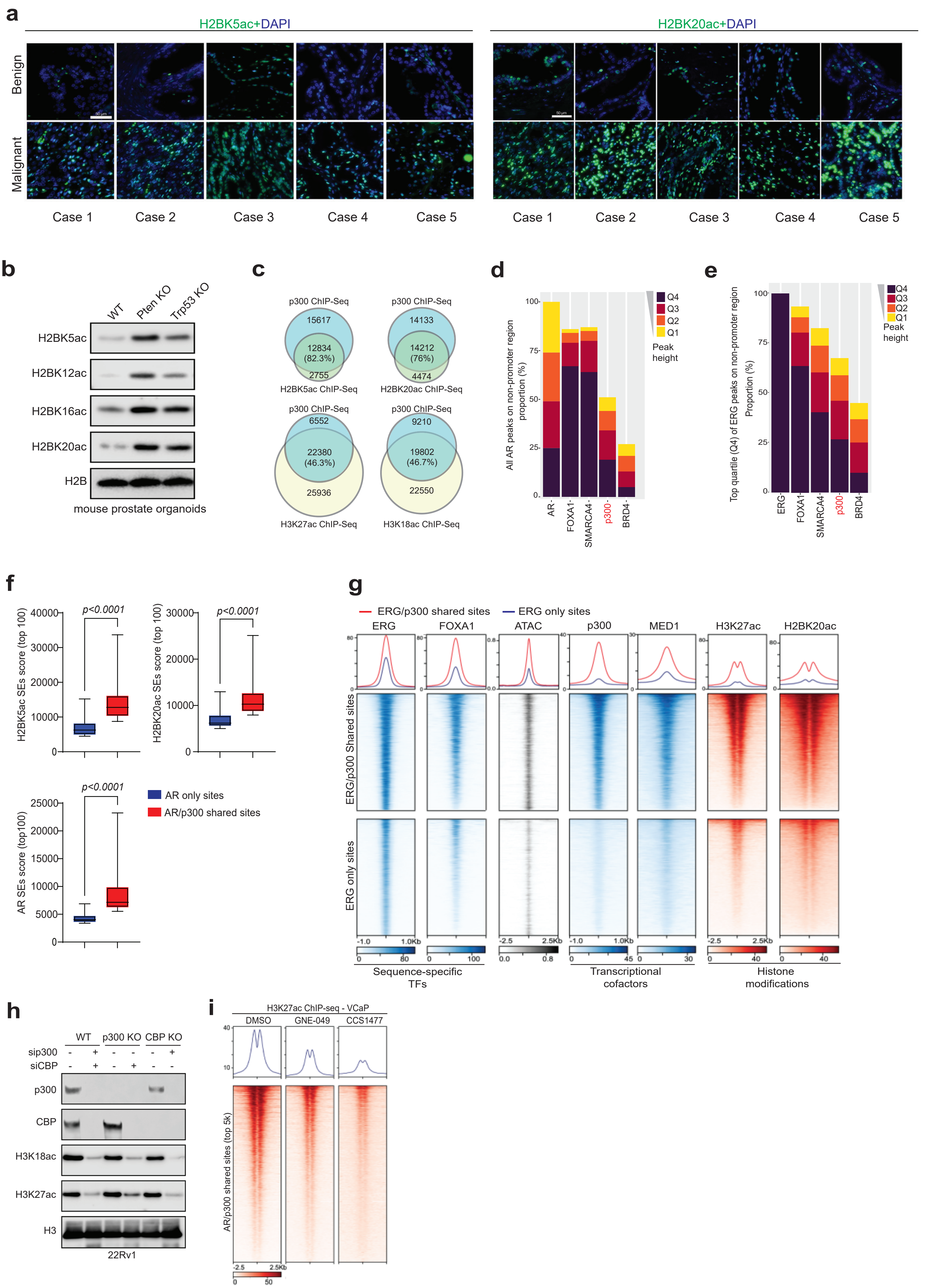

Extended Data Fig. 2

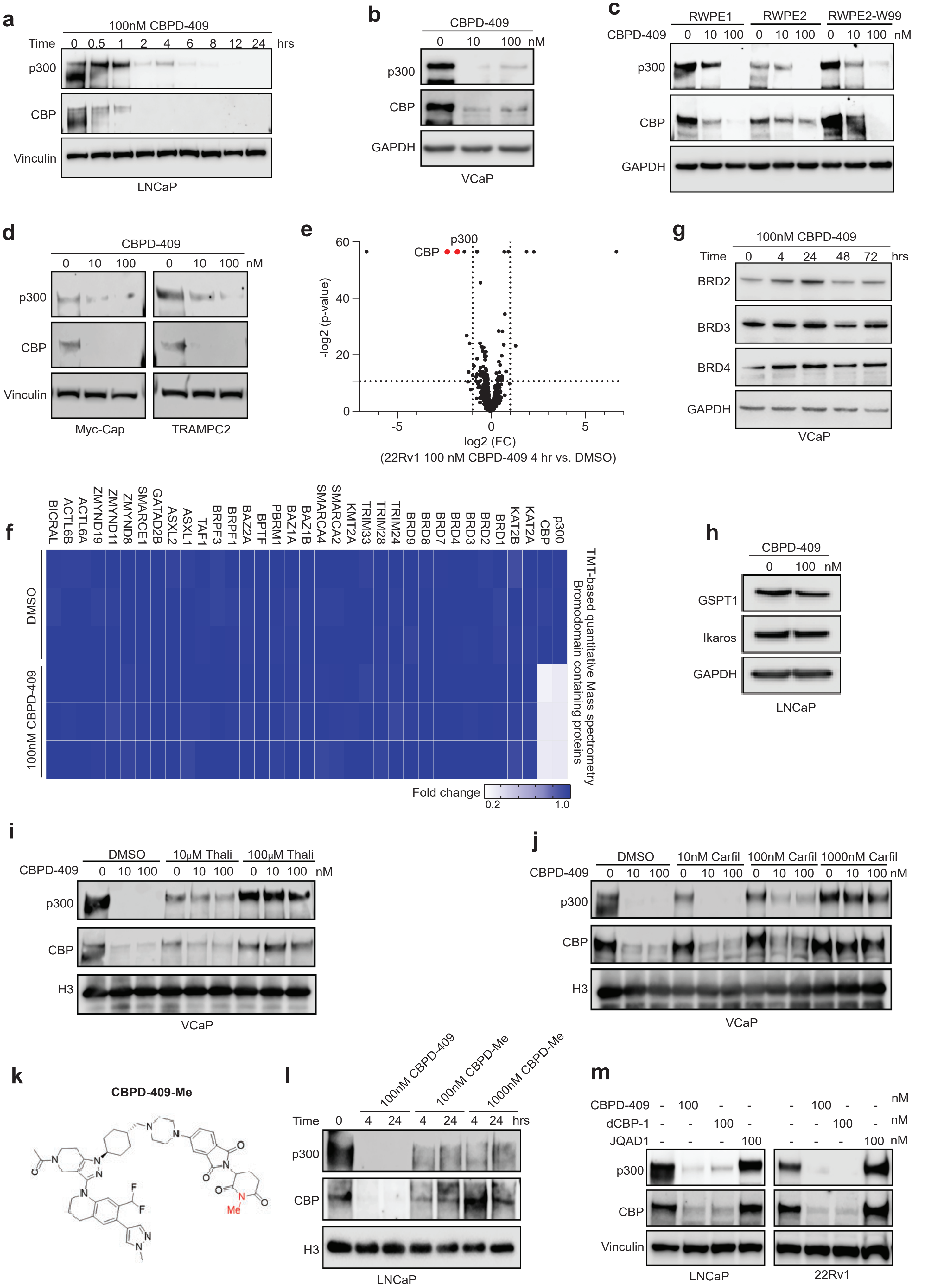

Extended Data Fig. 3

a

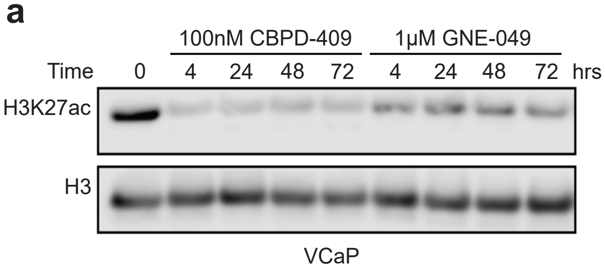

b

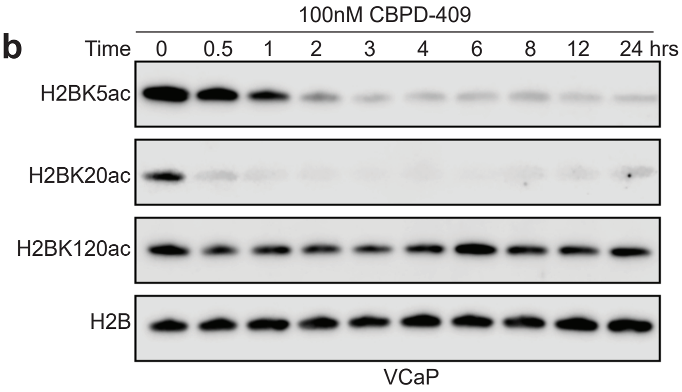

c

Summary of differential acetylated sites (acetylated proteins)

Group Name	Up-regulated (FC>1.5, p<0.05)	Down-regulated (FC<1/1.5, p<0.05)
DMSO vs CBPD 1hr	16 (11)	134 (80)
DMSO vs CBPD 4hrs	4 (2)	243 (151)
DMSO vs CBPD 12hrs	5 (5)	302 (197)
DMSO vs CBPD 24hrs	11 (8)	337 (214)

d

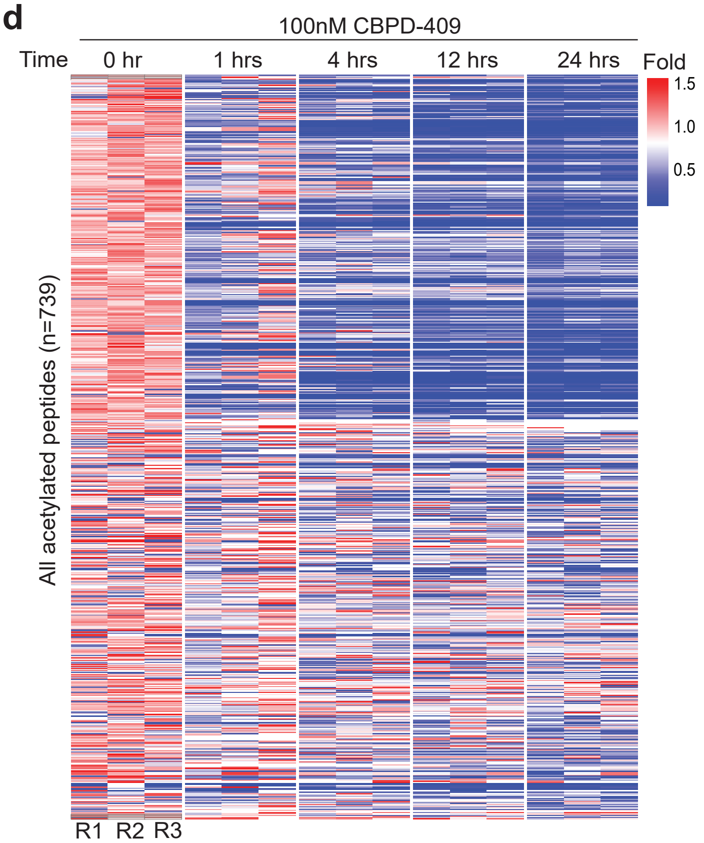

Extended Data Fig. 4

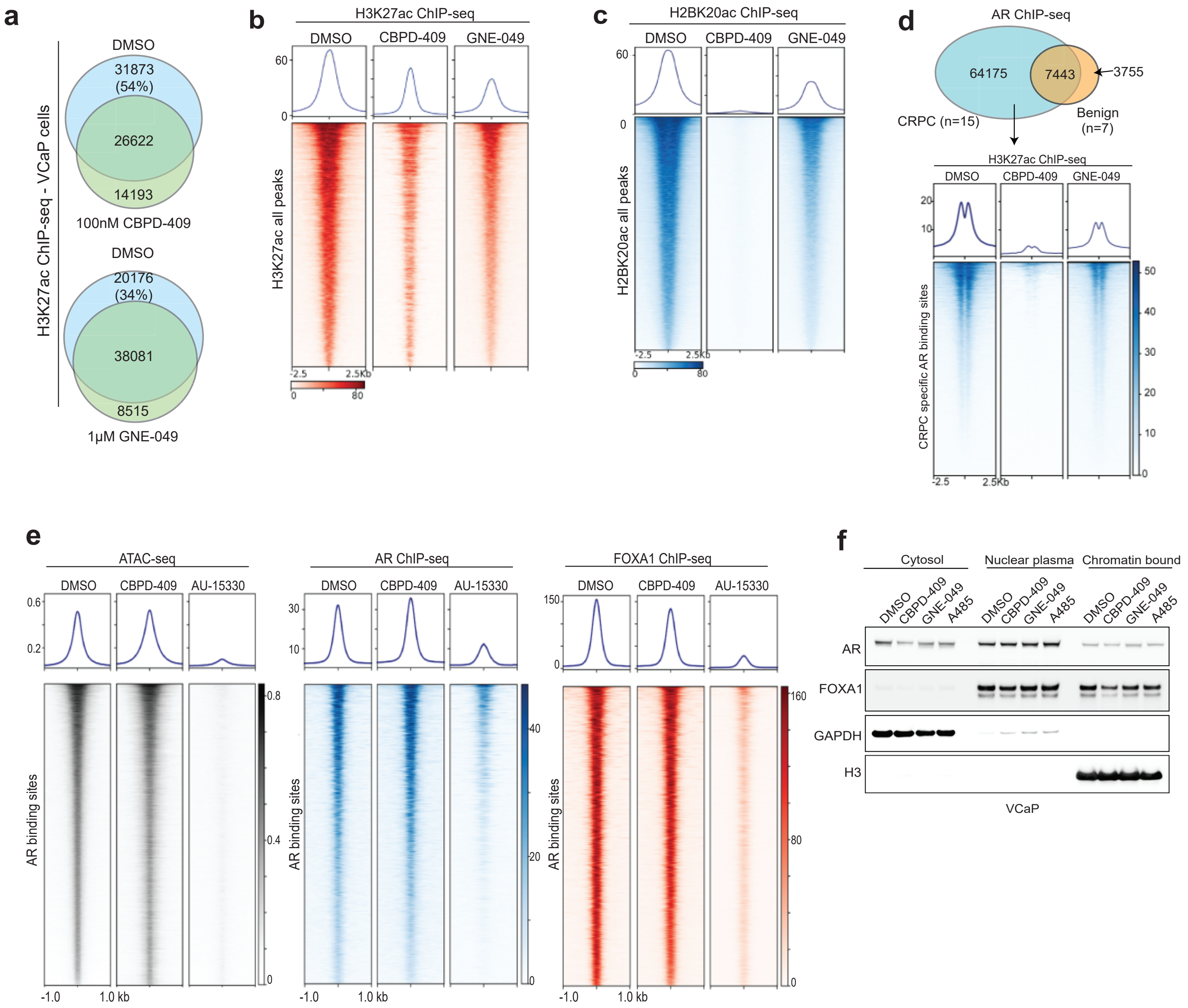

Extended Data Fig. 5

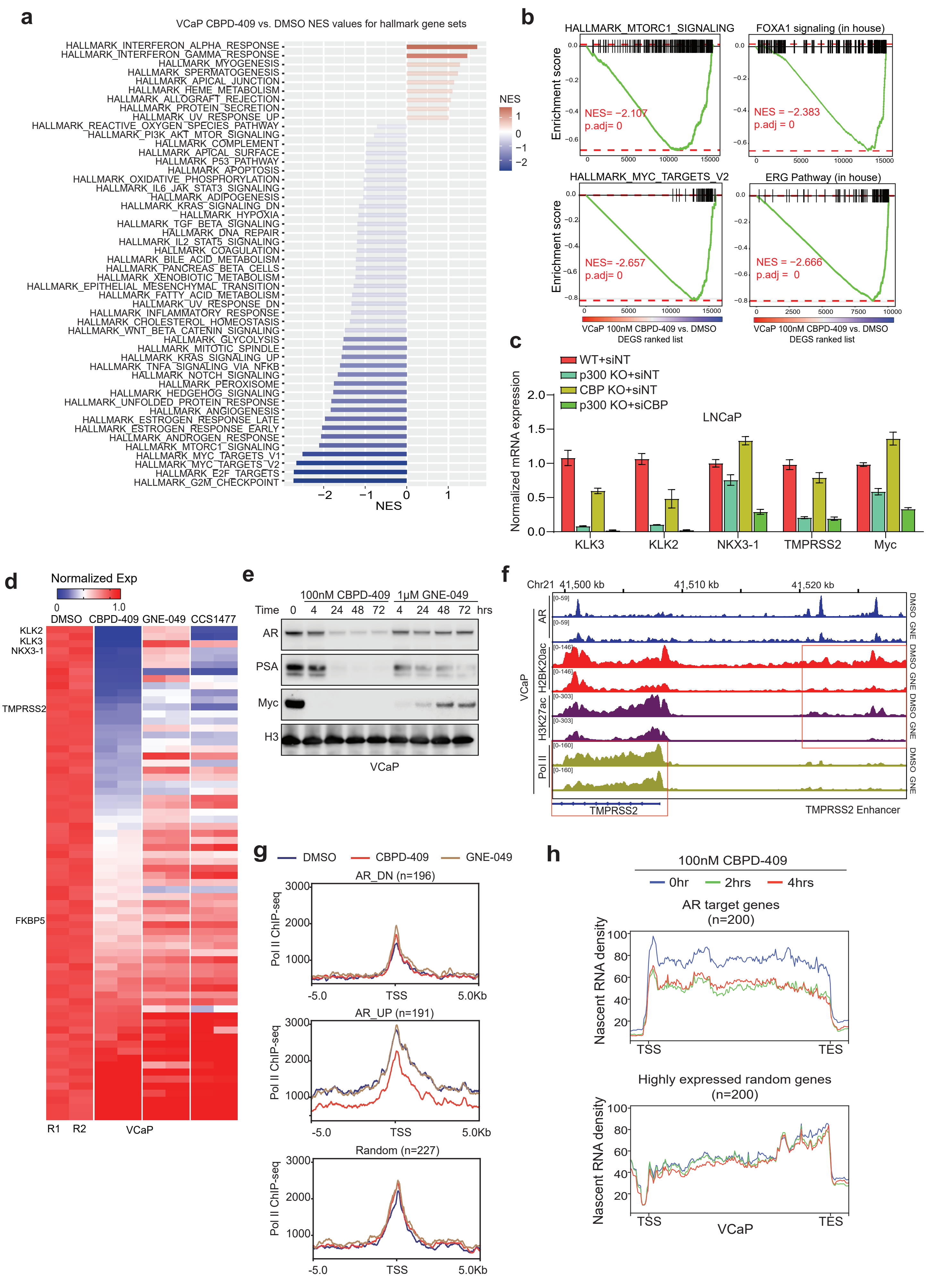

Extended Data Fig. 6

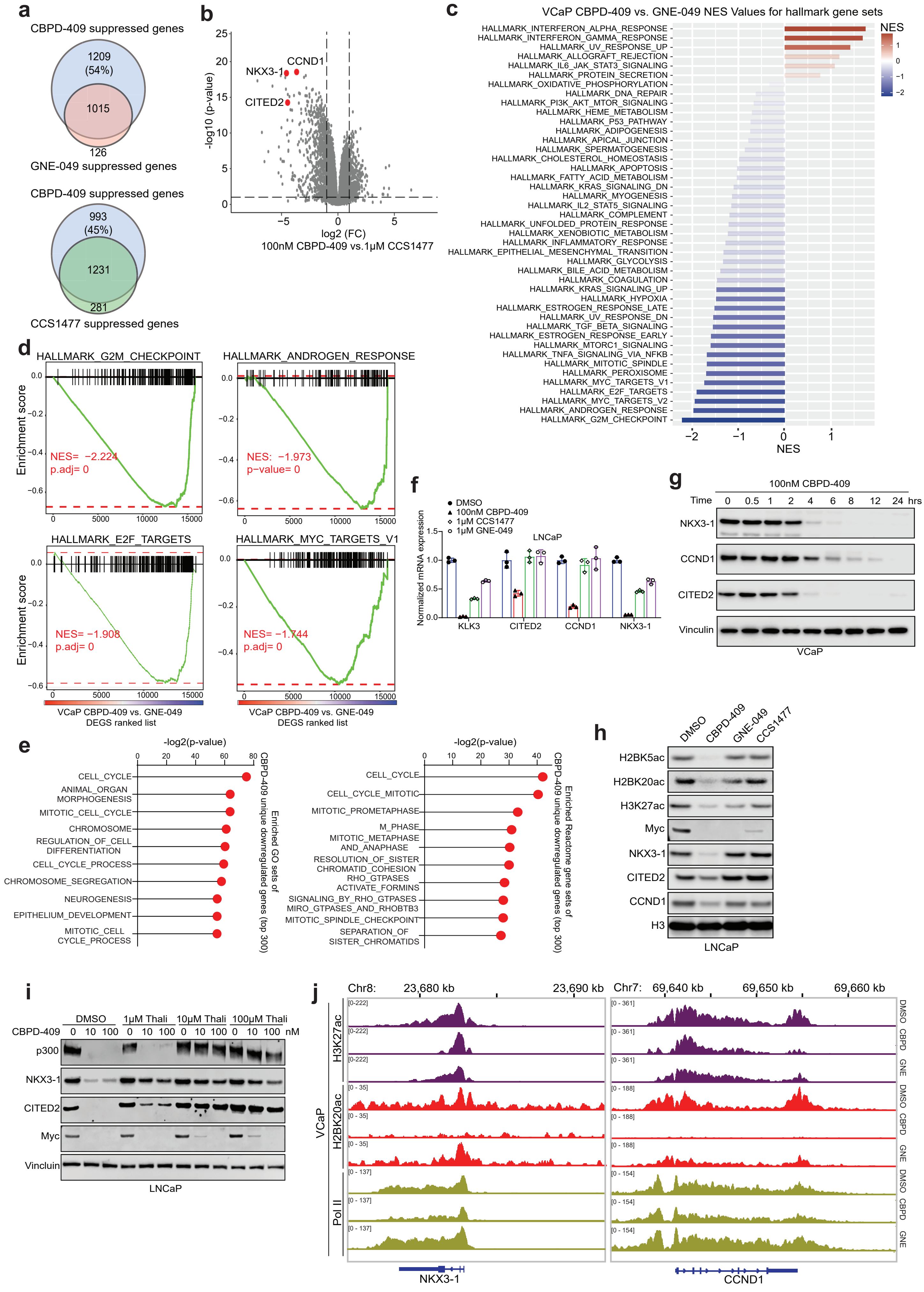

Extended Data Fig. 7

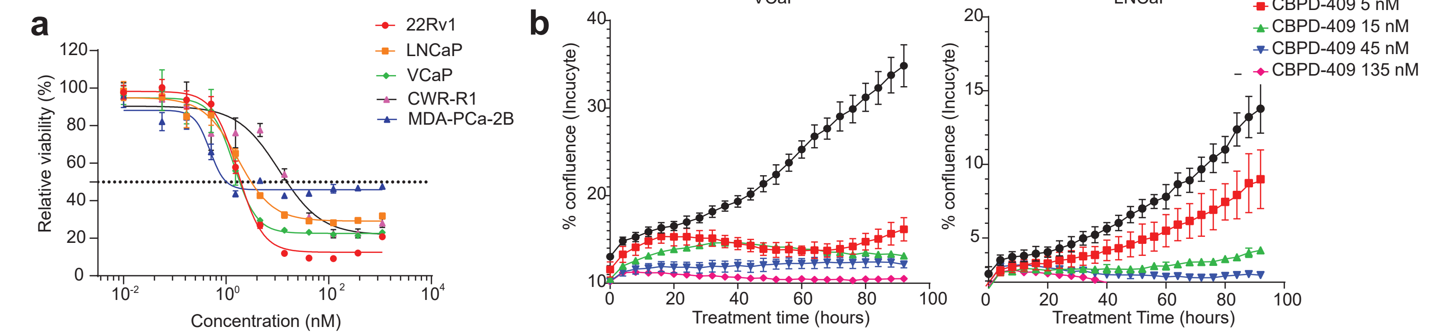

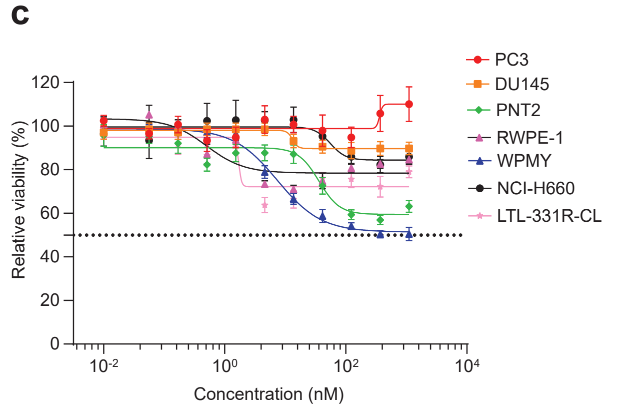

d

	Cell line	CBPD-409 IC50 (nM)
AR+ prostate cancer	22Rv1	2.2
	MDA-PCA-2B	2.5
	VCaP	4
	LNCaP	11
	CWR-R1	11.5
AR- prostate cancer	PC3	6368
	DU145	>30000
	NCI-H660	>30000
	LTL-331R-CL	>30000
non-neoplastic	WPMY-1	4365
	PNT2	>30000
	RWPE-1	>30000

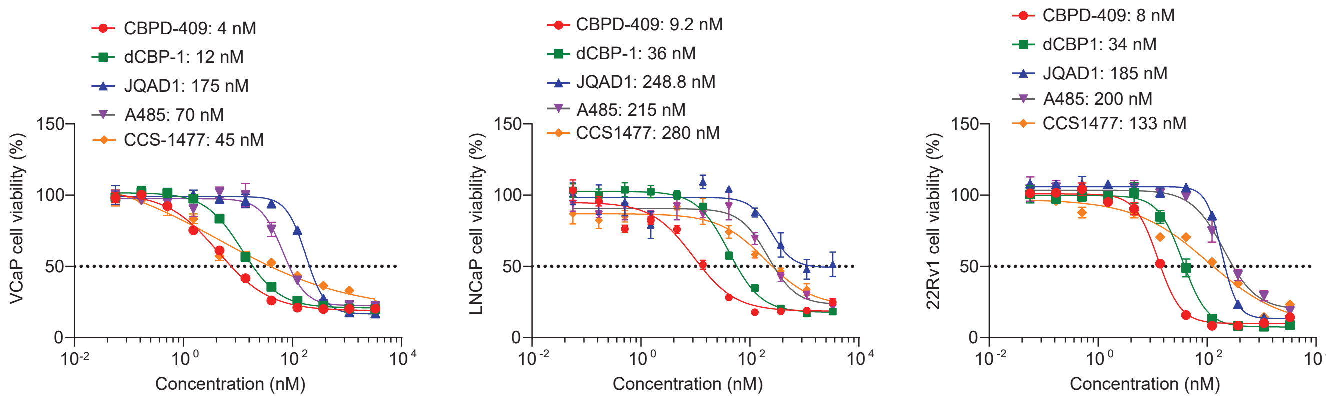

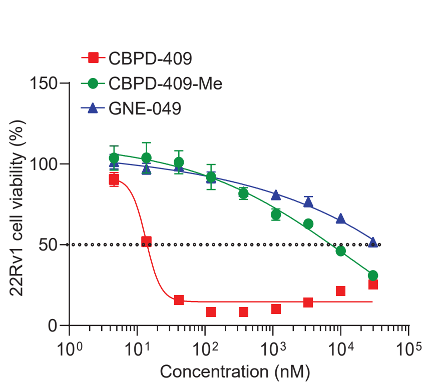

Extended Data Fig. 8

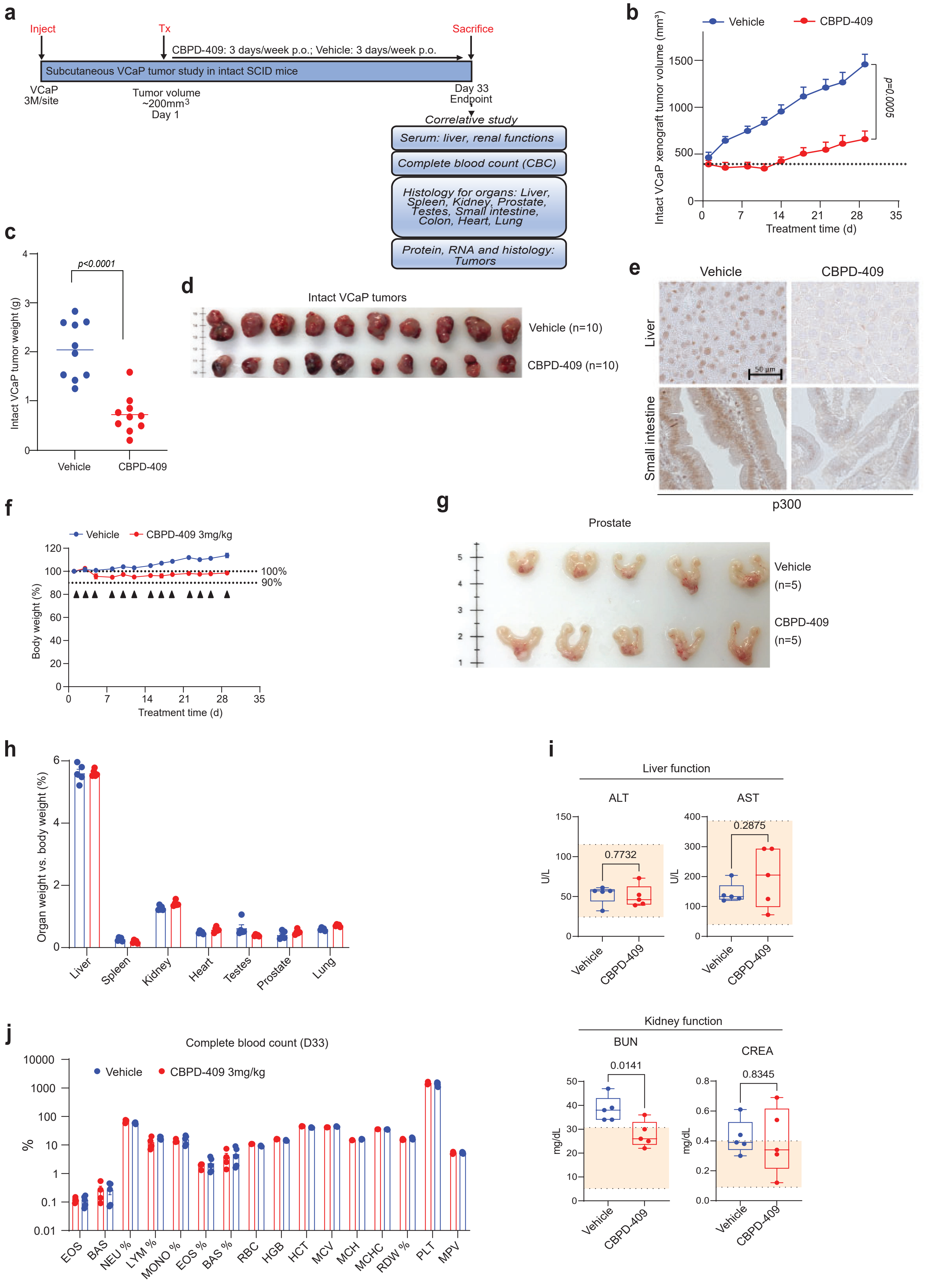

Extended Data Fig. 9

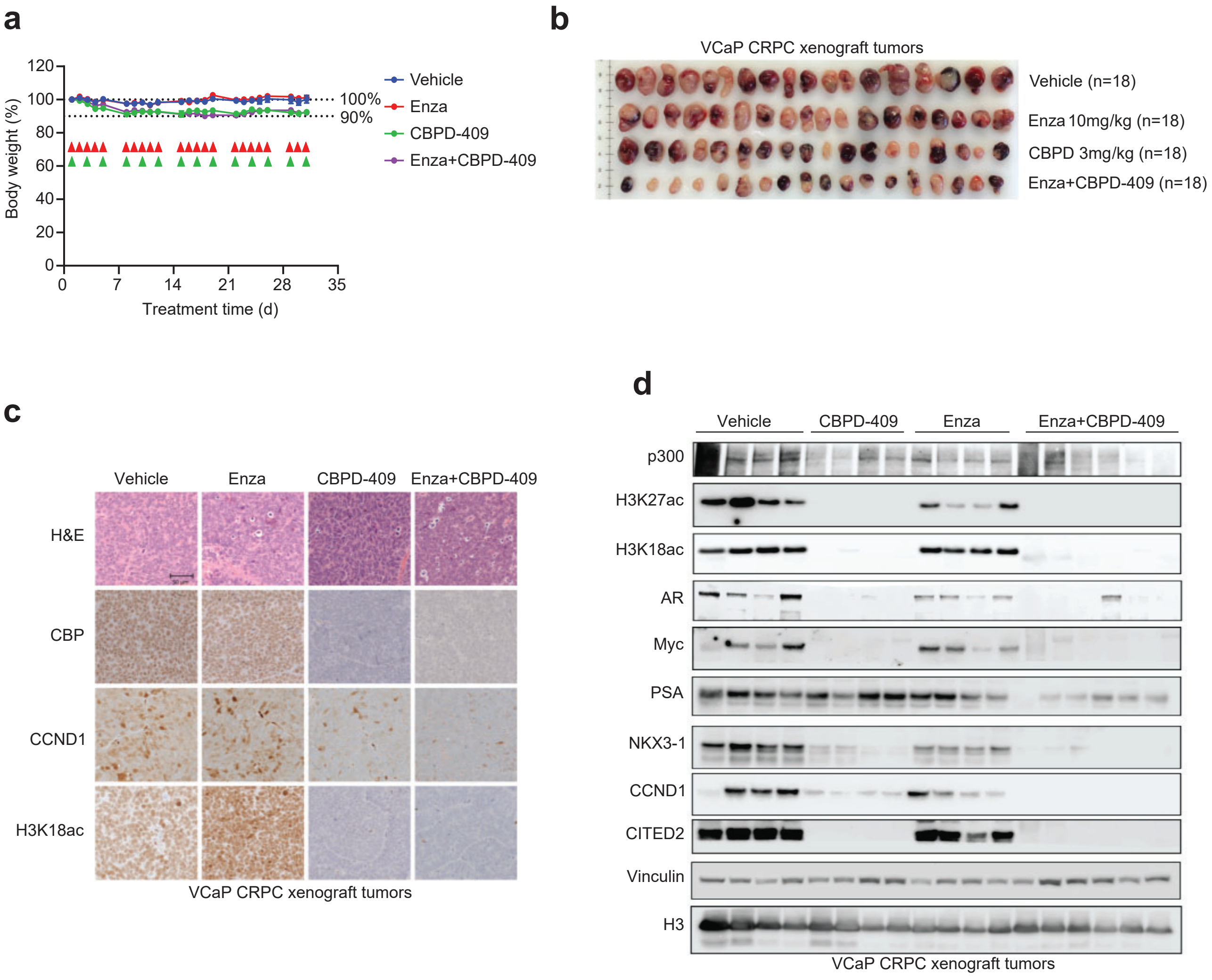

Extended Data Fig. 10

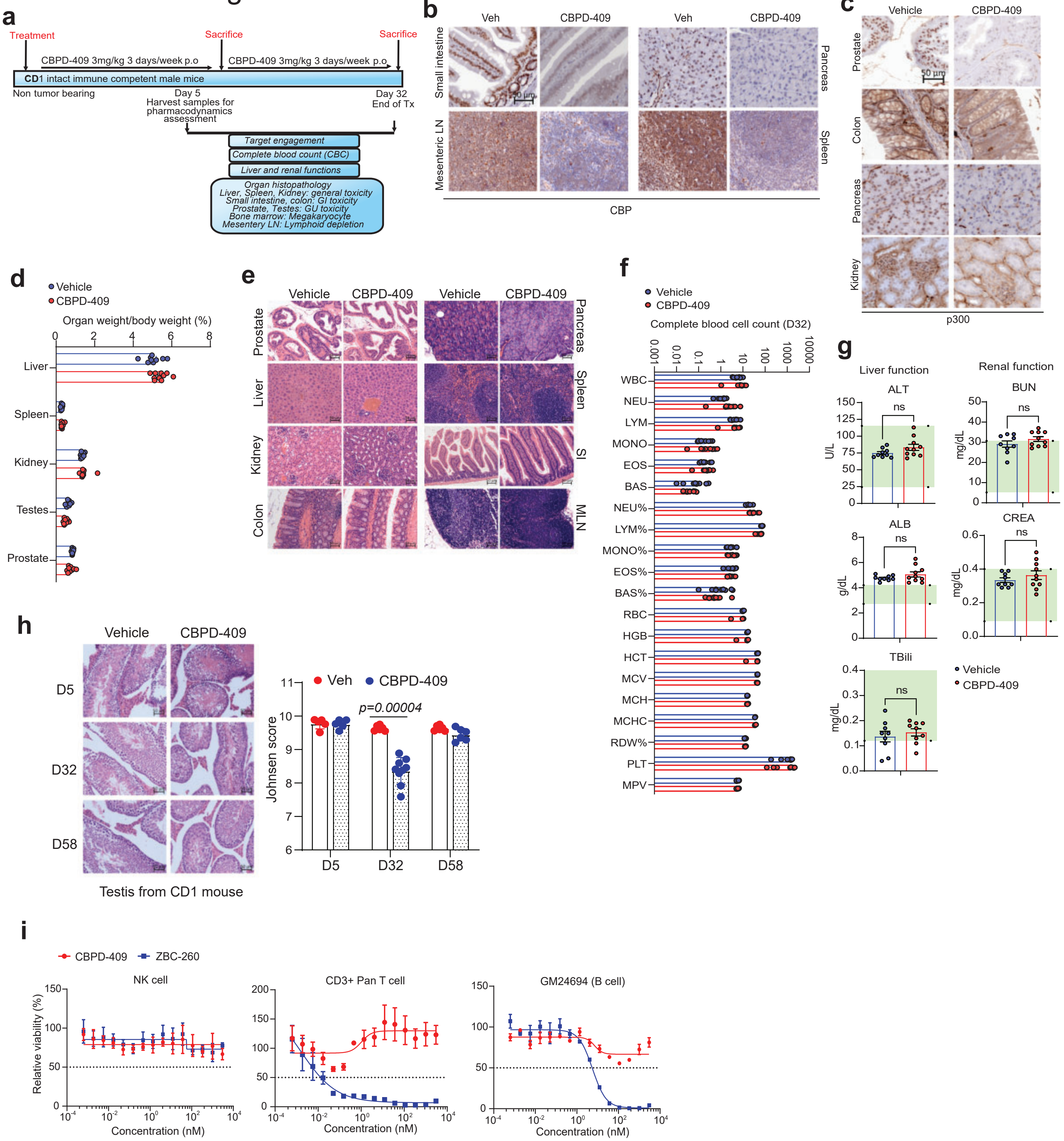

Extended Data Fig. 11

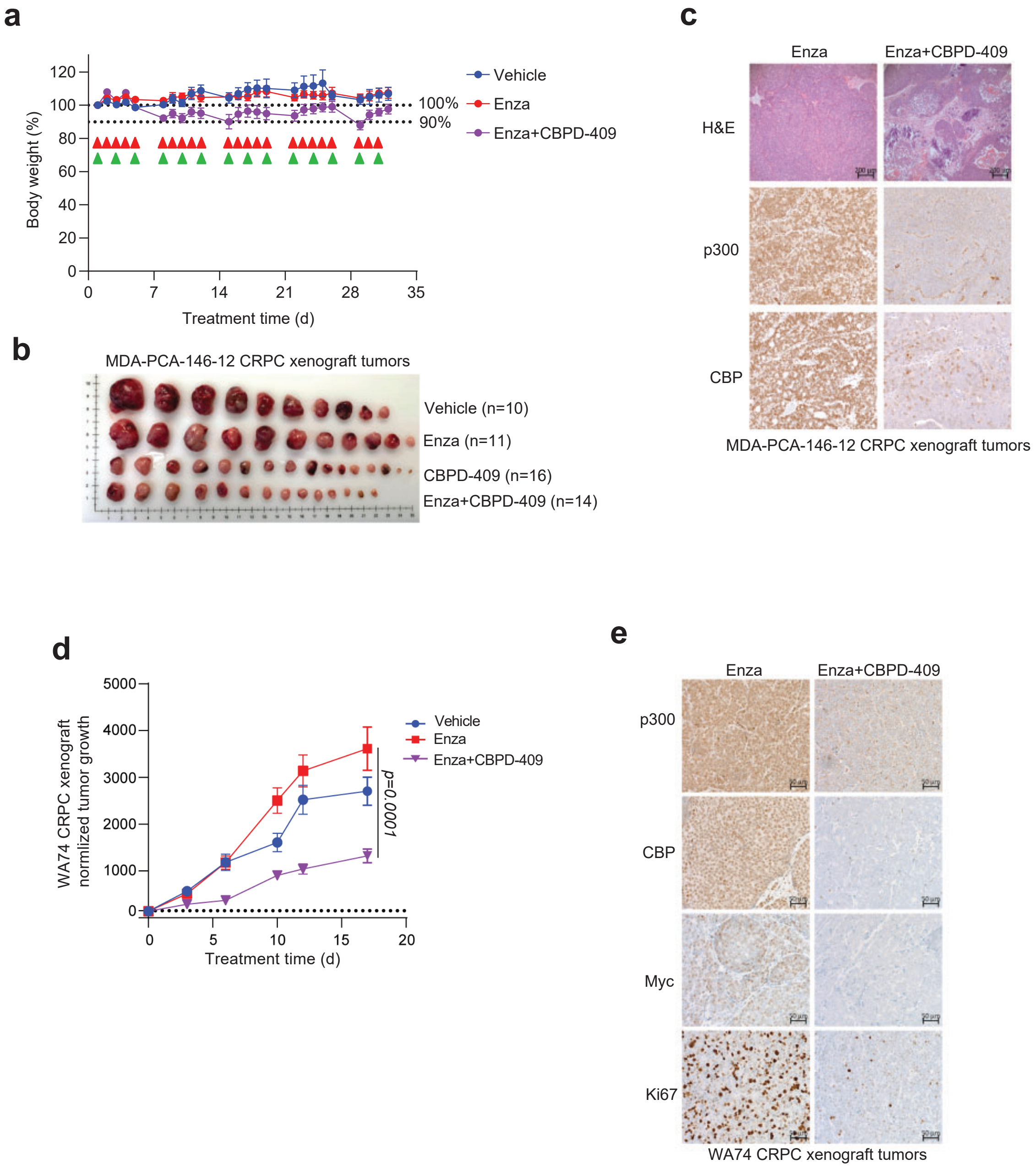

Supplement: Supplementary file 1 [file media-1.pdf]
